# Imaging of a case of atrioventricular septal defect: The added value of using the third dimension in echocardiography

**DOI:** 10.1002/ccr3.7544

**Published:** 2023-06-13

**Authors:** Mahmoud Abdelnabi, Abdallah Almaghraby, Hoda Abdelgawad

**Affiliations:** ^1^ Internal Medicine Department Texas Tech University Health Science Center Lubbock Texas USA; ^2^ Cardiology Department Ibrahim Bin Hamad Obaidullah Hospital Ras Al Khaimah UAE; ^3^ Cardiology Department, Faculty of Medicine Alexandria University Alexandria Egypt; ^4^ Cardiology Department King's college hospital NHS Trust London UK

**Keywords:** atrioventricular septal defect, congenital heart diseases, echocardiography, third dimension

## Abstract

The role of 3D echocardiography has been increasing in the management of patients with congenital heart disease (CHD), particularly in pre‐surgical planning, catheter‐guided interventions, and functional assessment of the heart.

## 1 CASE PRESENTATION

**FIGURE 1:**
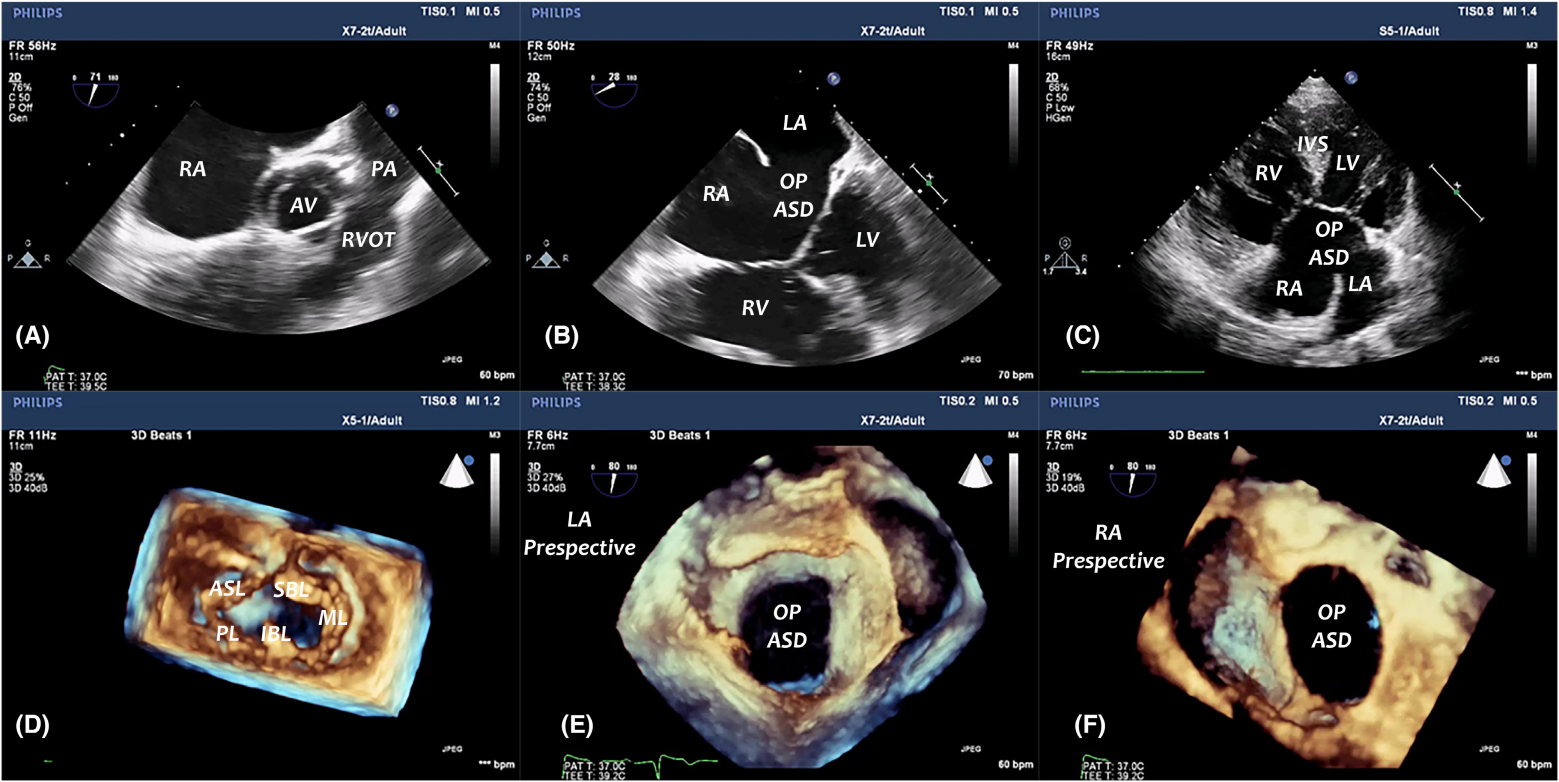
(A): 2D TOE (RV inflow‐outflow view) shows marked RVOT hypertrophy. (B, C): 2D TOE and TTE (Four chamber views) show a large ostium primum ASD. (D): Zoomed mode of the AV valve from the ventricular perspective confirms the presence of a common AV annulus with five leaflets (superior and inferior bridging leaflets, left mural leaflets, and anteroseptal and posterior leaflets). (E, F): Zoomed mode of the IAS from both left and right atrial perspectives shows an oval‐shaped ostium primum ASD.

A 24‐year‐old male patient presented complaining of a 4‐year history of worsening exertional dyspnea and easy fatigability, with no previous history of chest pain, palpitations, or episodes of cyanosis reported. On cardiac examination, a loud systolic murmur was heard over the left parasternal area. His electrocardiogram (ECG) showed an incomplete right bundle branch block (RBBB) while his Chest X‐ray showed cardiomegaly. 2D Transthoracic Echocardiography (2D TTE) revealed a large atrial septal defect (ASD), a cleft in left and right atrioventricular (AV) valves with mild regurgitation, a small restrictive ventricular septal defect (VSD), and marked right ventricular (RV) free wall hypertrophy with a systolic gradient across RV outflow tract (RVOT) of 50 mmHg. 3D Transesophageal echocardiography (TOE) with zoomed and full volume mode acquisition revealed a common AV valve with a common annulus with clear visualization of the five leaflets. The left AV valve cleft was directed toward the interventricular septum (IVS) with a small coaptation gap seen at the central orifice. An oval‐shaped large ostium primum ASD was seen from both right and left atrial perspectives, and multiple small fenestrated VSDs were seen in the inlet septum. Associated infundibular hypertrophy was noted with normal pulmonary valve orifice (Arrow) (Figure 1, Video S1, Video S2). So, the authors report a complete AVSD case associated with infundibular stenosis. She was referred for surgical AV valve repair, ASD and VSDs closure, and RVOT reconstruction. The role of 3D echocardiography has been increasing in the management of patients with congenital heart disease (CHD), particularly in pre‐surgical planning, catheter‐guided interventions, and functional assessment of the heart.^1^ Although being one of the common defects encountered, AVSD‐associated defect imaging remains challenging. Residual cleft and valve dysplasia are the main causes of reoperations.^2^ Although 2D echocardiography is the standard imaging modality used in AVSD evaluation, there are several limitations to 2D techniques that can be improved using 3D imaging. 3D allows depth perception and is superior in detecting leaflet and anterior commissural abnormalities, complex AV valve, and LVOT abnormalities associated with AVSD. It can also provide additional information regarding the mechanisms and sites of left AV valve (LAVV) regurgitation and proper estimation of the numbers of regurgitation jets in AVSDs compared with 2D techniques.^3^

## AUTHOR CONTRIBUTIONS

**Mahmoud Abdelnabi:** Writing – original draft; writing – review and editing. **Abdallah Almaghraby:** Writing – original draft; writing – review and editing. **Hoda Abdelgawad:** Writing – original draft; writing – review and editing.

## CONFLICT OF INTEREST STATEMENT

None declared.

## CONSENT STATEMENT

Patient consent has been signed and collected in accordance with the journal's patient consent policy.

## Supporting information


Video S1:

Video S2:

## Data Availability

All case‐related data are available as part of the article, and no additional source data are required.
